# Daylight saving time and mortality—proceed with caution

**DOI:** 10.1038/s41467-024-45837-4

**Published:** 2024-02-21

**Authors:** Elizabeth B. Klerman, Matthew D. Weaver, Till Roenneberg, Beth A. Malow, Karin G. Johnson

**Affiliations:** 1 Department of Neurology, Massachusetts General Hospital, Harvard Medical School, Boston, MA USA; 2 Division of Sleep Medicine, Harvard Medical School, Boston, MA USA; 3 Departments of Medicine and Neurology, Division of Sleep and Circadian Disorders, Brigham and Women’s Hospital, Boston, MA USA; 4 Institute for Occupational-, Social-, and Environmental Medicine, LMU Munich, Munich, Germany; 5 Institute for Medical Psychology, LMU Munich, Munich, Germany; 6 Chronsulting, Priel, Germany; 7 Sleep Disorders Division, Department of Neurology, Vanderbilt University Medical Center, Nashville, TN USA; 8 Department of Neurology, Baystate Medical Center, University of Massachusetts Chan Medical School-Baystate, Springfield, MA USA; 9 Department of Healthcare Delivery and Population Science, University of Massachusetts Chan Medical School-Baystate, Springfield, MA USA

**Keywords:** Epidemiology, Risk factors, Circadian rhythms and sleep

**arising from** L. Lévy et al. *Nature Communications* 10.1038/s41467-022-34704-9 (2022)

The article *“Daylight saving time affects European mortality patterns”* by Lévy et al.^1^ reports associations between all-cause mortality and multiple factors, including the clock time changes between standard time [ST] and daylight saving time [DST] in spring and fall, using data from 1998 and 2012 in 16 European countries. While the abstract reports that mortality decreases for two weeks after the spring change and shows a reverse effect in the fall, Figure 1b/c clearly shows the opposite in the weeks after the transitions: a relative *increase* from already decreasing mortality trend after the spring change and a relative *decrease* from the already increasing trend in the fall. The authors base their abstract text on the outcome of a multiple negative binomial regression model. Here, we suggest major flaws in the statistical model and conclusions. In addition, the title and conclusions suggest that the findings can be linked to long-term DST effects; this misattribution may lead people to believe these data inform the choice between permanent DST or permanent ST—which they do not.

The data in Figure 1 are consistent with multiple previous reports, including some listed in the source article^2–4^. Why does their regression model (Table 1) reverse the direction of the results from Figure 1? We present five likely reasons:

1. Severe concurvity in the statistical model (note that “concurvity” is used in non-linear models and “collinearity” in linear models). The most relevant concurvity parameters used are season, month, DST transition weeks, a sinusoid representing photoperiod, temperature, and humidity. All of these parameters are interdependent, yet the authors do not mention testing for concurvity and potentially adjusting the model appropriately.
2. Table 1 is an excellent (though not exhaustive) compilation of parameters that are associated with mortality rates. Since the stated focus of this article is whether DST transitions are associated with changes in mortality rates, the statistical model used (even if there are no concurvity problems) is not correct. An appropriate model would test whether the parameters listed in Table 1 affect mortality rates associated with the DST transitions (e.g., whether the observed results are only specific for a given year or whether the age or sex composition differs before and after DST clock transitions).
3. Since the research question is about mortality associated with DST transitions, the dataset should only include weeks of data before and after those transitions, not the entire time series. This smaller dataset would also have fewer covariates. Note that the authors describe the relevant time as 2 months before and after the DST clock transitions, yet the model includes data from throughout the year. This may introduce bias in the predicted IRR estimates that are used as the referent, since the covarying seasonal variations (e.g., photoperiod, temperature, month, humidity) proximal to the referent time intervals interact with, and are attenuated by the longer-term trends, included in the model (as in comment 1). An analogy would be the investigation of why someone died in a traffic accident: relevant conditions are the characteristics of the victim, time-of-day, weather, and traffic density for some time around the accident—not across the entire year.
4. Although the paper’s Figure 1 shows data from 2 months before and after the DST changes, the statistical model’s comparison for DST results is based on a single week before the changes, which is not appropriate given the longer-term seasonal trends in the data (e.g., rising in fall, falling in spring). These seasonal trends alone cause the mortality to be higher a few weeks in the fall and lower in the spring after the comparison week—as is found using this statistical model. This method does not unveil the independent effect of DST transitions. When a comparison is made vs. this trend (as in Figure 1b/c), the opposite change in mortality rates is seen. Therefore, incorporating seasonal trends explicitly is critical—e.g., through an Interrupted Time Series design using a carefully defined pre-post interval that does not include the entire year to test a change in slope.
5. The authors have a mixed design with 16 countries observed from 1998 to 2012. Whether or not this is adequately accounted for in the pooled regression model is uncertain. The authors do not test interactions between countries and the variables of interest. Ignoring the existence of more than one dimension, as in this case, leads to biased estimators and wrong variances of the estimators.

Other considerations

1. The model is constructed to include many parameters potentially associated with mortality risk and report the relative importance of each. The incidence rate ratio (IRR) reported as statistically significant for the spring is 0.97 (weeks 1 and 2) and for the fall is 1.02 (weeks 1, 2)—and, contrary to their conclusions, 0.99 in spring week 0 and 0.97 in fall week 4. These differences probably reflect the variation in the data. Any statistical significance is likely achieved due to the massive sample size. While even one excess death is too many, the relative impact of DST transitions in this analysis is very low. The IRRs for other metrics are similar (e.g., daily humidity) or much higher (e.g., year, month, age, country). These other statistically significant factors do not receive the same emphasis by authors—exemplified by the misleading title of the paper. In addition, more commonly used adjustments for multiple comparisons would render the primary finding as mixed or non-significant (e.g., Bonferroni adjustment would have *P* value = 0.0006), despite the robust sample size.
2. Since the reason for deaths is not reported, no distinction can be made between deaths expected to be related to DST transitions (e.g., traffic accidents) vs. those that have longer-term determinants (e.g., cancer).
3. Even without the potential statistical issues mentioned above, the over-extension of these results (of weeks after each transition) to claim that DST (which is present for many months of the year) affects mortality is not supported and should not be included in the title or in the discussion.

The paper’s results are inconsistent with multiple other studies of health effects associated with DST transitions. The spring ST- > DST transition is associated with increases in the most common causes of death—heart attacks^2^ and strokes^4^. In addition, there are long-term adverse health effects due to disturbances in sleep and circadian rhythm misalignment throughout DST that are independent of any short-term transition effects^5–7^. Introducing DST is equivalent to making people live according to the local clock time of one time zone further east (i.e., Chicago residents must live on Boston time). The underlying mechanisms of the sleep and circadian effects are chronic, and include dysregulation of melatonin and cortisol contributing to stress, altered metabolism, and inflammation^8,9^. DST also has adverse effects on neuropsychological function^10^ that are associated with (i) increases in suicides^11^ and traffic accidents^12^, despite short-term benefits related to light exposure during evening commutes, and (ii) more irregular adolescent sleep times and increased seasonal depression^13^.

Data from multiple sources led many scientific and sleep societies around the world to conclude that the health risks of permanent DST would be worse than both the status quo (i.e., changing between DST/ST) and permanent ST^8,9,14,15^. We support the positions of these societies and advocate for permanent ST.
